# Tricycloalternarene Analogs from a Symbiotic Fungus *Aspergillus* sp. D and Their Antimicrobial and Cytotoxic Effects

**DOI:** 10.3390/molecules23040855

**Published:** 2018-04-09

**Authors:** Huawei Zhang, Ziping Zhao, Jianwei Chen, Xuelian Bai, Hong Wang

**Affiliations:** 1 School of Pharmaceutical Sciences, Zhejiang University of Technology, Hangzhou 310014, China; hwzhang@zjut.edu.cn (H.Z.); marinedrugs2017@163.com (Z.Z.); cjw983617@zjut.edu.cn (J.C.); 2 College of Life and Environmental Sciences, Hangzhou Normal University, Hangzhou 310038, China; baixl2012@163.com

**Keywords:** tricycloalternarene, meroterpenoid, *Aspergillus* sp., *Edgeworthia chrysantha* Lindl., symbiotic microbe, coastal plant

## Abstract

Bioassay-guided fractionation of the crude extract of fermentation broth of one symbiotic strain *Aspergillus* sp. D from the coastal plant *Edgeworthia chrysantha* Lindl. led to isolation of one new meroterpenoid, tricycloalternarene 14b (**1**), together with four known analogs (**2**–**5**), tricycloalternarenes 2b (**2**), 3a (**3**), 3b (**4**), and ACTG-toxin F (**5**). Their chemical structures were unambiguously established on the basis of NMR, mass spectrometry, and optical rotation data analysis, as well as by comparison with literature data. Biological assays indicated that compound **2** exhibited potent in vitro cytotoxicity against human lung adenocarcinoma A549 cell line with an IC_50_ value of 2.91 μM, and compound **5** had a moderate inhibitory effect on *Candida albicans*, with an MIC value of 15.63 μM. The results indicated that this symbiotic strain D is an important producer of tricycloalternarene derivatives, with potential therapeutic application in treatment of cancer and pathogen infection.

## 1. Introduction

The fungi of the *Aspergillus* genus are well known to be a rich source of secondary metabolites with a broad spectrum of biological activities. The *Aspergillus* Secondary Metabolites Database (A2MDB) had documented 807 unique non-redundant natural products derived from 675 *Aspergillus* species by 2017 [1]. A growing amount of evidence also indicates that symbiotic *Aspergillus* strains are an important contributor of bioactive natural products. By 2014, up to 162 new bioactive chemicals had been characterized from 11 *Aspergilli* spp. as endophytic microbes [2]. Tricycloalternarenes (TCAs) represent a group of fungal-derived meroterpenoids, and are produced by several genera, including *Aspergillus* [3], *Alternaria* [4,5,6], *Guignardia* [7], *Ulocladium* [8], etc. Structurally, TCAs are closely related to ACTG-toxins. The differences mainly occur in the isoprenoid side chain and the substitution pattern of the C-ring of TCAs. These metabolites are of intense interest to natural drug chemists due to their remarkable antimicrobial and cytotoxic effects.

**Figure 1:**
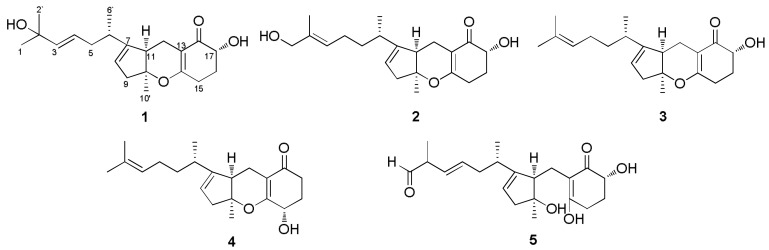
Chemical structures of compounds **1**–**5**.

In our ongoing chemical investigation of symbiotic *Aspergillus* strains for discovery of new bioactive natural products, one new meroterpenoid, named TCA 14b (**1**), along with four derivatives, TCAs 2b (**2**), 3a (**3**), 3b (**4**), and ACTG-toxin F (**5**), was isolated from *Aspergillus* sp. D associated with the coastal plant *Edgeworthia chrysantha* Lindl (Figure 1). Details on the isolation and structural elucidation of these compounds are reported in this paper, as well as the results of cytotoxic and antimicrobial assays.

## 2. Results and Discussion

Bioassay-guided fractionation of the EtOAc crude extract of fermentation broth of the strain D led to isolation of five compounds (**1**–**5**) using the semi-preparative and analytical HPLC approach. The chemical structures of compounds **2**–**5** were assigned as TCAs 2b (**2**), 3a (**3**), 3b (**4**) and ACTG-toxin F (**5**) based on spectroscopic comparisons with literature data [9,10,11,12,13]. A structure argument for the new compound **1** is described below.

**Figure 2:**
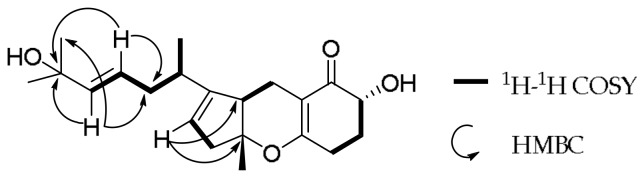
Key ^1^H−^1^H COSY and HMBC correlations of **1**.

Compound **1** was obtained as yellowish amorphous powder. Its positive HR-ESI-MS at *m*/*z* 369.2039 [M + Na]^+^ indicated a molecular formula of C_21_H_30_O_4_ with 7 degrees of unsaturation (calcd. for C_21_H_30_O_4_Na 369.2036), which is isomeric to TCA 2b (**2**). The ^1^H-NMR spectrum of **1** showed a good agreement with that of compound **2** (Supplementary Material). In order to facilitate structure elucidation, the ^1^H and ^13^C-NMR data for **1** and **2** are listed in Table 1. Careful inspection of proton NMR, HSQC and COSY spectra suggested that compound **1** has a [5,6,6] heterocyclic moiety and one 2-methyl-heptenic group at C-7. In contrast to **2**, however, compound **1** possesses one more olefinic proton (δ_H_ 5.50) and one more methyl (δ_H_ 1.29). It was deduced that the double bond between C-2 and C-3 in **2** was transferred to the position of C-3 and C-4 in **1**, while the hydroxyl group at C-1 in **2** was moved to C-2 in **1**. This assumption was certified by the observed variation of their ^13^C-NMR data, in which the chemical shift values of C-1 (68.7), C-2 (135.2) and C-4 (24.9) in **2** were changed to 29.8, 70.7, 125.2, respectively, in **1** (Table 1). The planar structure of **1** was further verified by its key ^1^H-^1^H COSY and HMBC correlations from H-3 to C-2, C-2′ and C-5, and from H-4 to C-2, C-3 and C-5 (Figure 2).

**Figure 3:**
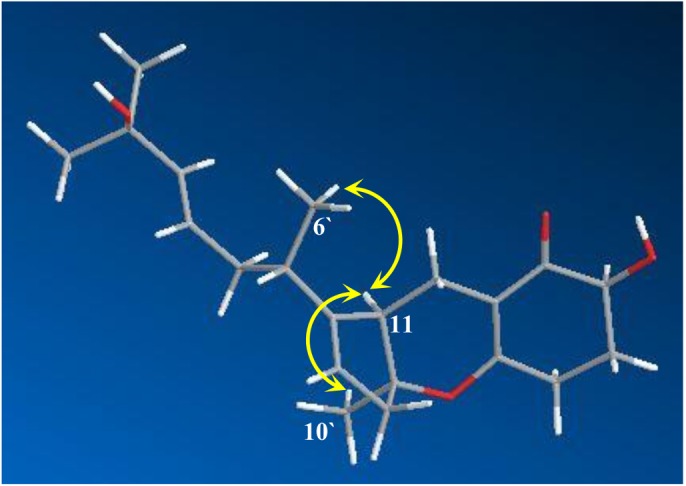
Strong NOESY correlations observed in **1**.

The configuration of the double bond between C-3 and C-4 in compound **1** was determined as *E* based on its ^3^*J*_3__–4_ value of 15.6 Hz. Its relative configuration was established on the basis of the important NOESY correlations of H-11 to H_3_-6′ and H_3_-10′ (Figure 3) and the optical rotation (${\left[\alpha \right]}_{D}^{25}$ = +78°), which was similar to that of compound **2**. Hence, **1** was identified as a new natural product and named as TCA 14b.

All isolated metabolites (**1**–**5**) were subjected to antimicrobial evaluation on three human pathogenic strains (*Escherichia coli*, *Staphyloccocus aureus* and *Candida albicans*) and preliminary cytotoxicity screening in vitro using CCK-8 assay against human lung carcinoma A-549 cell. Bioassay results showed that compounds **1**–**5** exhibited selective inhibitory effects on *E. coli*, *S. aureus*, *C. albicans* and A-549. Only ACTG-toxin F (**5**) clearly inhibited the growth of *C. albicans*, with an MIC value of 15.63 μM, whereas weak inhibition was observed for other compounds (MIC ≥ 31.25 μM). Compounds **1**–**5** possessed a moderate cytotoxic effect against human lung carcinoma A-549, with IC_50_ values of 8.89, 1.43, 10.35, 2.90, and 15.77 μM, respectively.

## 3. Experimental Section

### 3.1. General Experimental Procedures

NMR spectra were recorded on a 500 MHz Bruker Avance DRX500 spectrometer (Bruker, Fällande, Switzerland) equipped with a 5 mm triple resonance (HCN) cold probe, using TMS as an internal standard. Melting points were measured on a XRC-1 apparatus (Sichuang University Science and Education Instrument Factory, Chengdu, China) and were uncorrected. Optical rotations were obtained on a JASCO P-2000 polarimeter (JASCO, Fukuoka, Japan). Ultraviolet (UV) spectra were recorded on Hitachi-UV-3000 spectrometer (Hitachi, Tokyo, Japan), and FT-IR spectra were determined on Nexus 870 spectrometer (Thermo Nicolet, Madison, WI, USA). ESI-MS and HR-ESI-MS data were taken on an Agilent 6210 LC/TOF-MS spectrometer (Agilent Technologies, Santa Clara, CA, USA). Purification of all compounds was performed on Waters D600 apparatus (Waters, San Diego, CA, USA) equipped with a semi-preparative column (YMC-PACK-ODS-A, 250 × 10 mm, 5 μm, YMC, Kyoto, Japan) and an analytical column (Synergi Hydro-RP, 250 mm × 4.6 mm, 4 μm, Phenomenex, Torrance, CA, USA). Acetonitrile (Merck, Darmstadt, Germany) and H_2_O used in HPLC system were of chromatographic grade, and all other chemicals were analytical.

### 3.2. Fungal Materials

Fungal strain D was isolated from the healthy leaves of the coastal plant *Edgeworthia chrysantha* Lindl., collected from Hangzhou Bay, China. The culture was grown on potato dextrose agar (PDA) and identified as *Aspergillus* sp. on the basis of its morphological characteristics and analysis of 18S rDNA gene sequence (GenBank accession No. KR019681). This fungus is maintained as a cryopreserved glycerol stock at School of Pharmaceutical Sciences, Zhejiang University of Technology, China.

### 3.3. Fermentation, Extraction, and Isolation

Strain D was cultured on PDA at 28 °C for 7 days. A balanced amount of fungal colony was transferred to culture broth in 500-mL Erlenmeyer flask, which contained 250 mL salted Czapek’s medium consisting of sucrose 30 g/L, NaCl 30 g/L, NaNO_3_ 3 g/L, K_2_HPO_4_ 1 g/L, KCl 0.5 g/L, MgSO_4_·7H_2_O 0.5 g/L, FeSO_4_ 0.01 g/L, followed by shaking at 200 rpm at 28 °C for 10 days. At the end of fermentation, all broth was collected and filtered through gauze, which afforded the filtrate (approximate 60 L). The filtrate was extracted twice with the same volume of ethyl acetate (Merck). The upper solvent was evaporated at 25 °C in vacuum to yield the extract (about 2.0 g) followed by separation on a semi-preparative HPLC column to afford six fractions (F1–F6) under an isocratic condition of 75% CH_3_CN in H_2_O with a flow rate of 3.0 mL/min and 260 nm detection. Then bioactive fraction F2 was further subjected to HPLC with an analytical HPLC column (1.0 mL/min) to give compounds **1** (2.5 mg, 0.125%), **2** (5.1 mg, 0.255%) and **5** (12.2 mg, 0.61%) under an isocratic condition of 60% CH_3_CN with a flow rate of 1.0 mL/min at 260 nm. Compounds **3** (7.2 mg, 0.36%) and **4** (4.5 mg, 0.225%) were respectively purified from bioactive fractions F3 and F6 using the same analytical column under an isocratic condition of 65% CH_3_CN.

Tricycloalternarene 14b (**1**): Yellowish amorphous powder; C_21_H_30_O_4_; m.p. 59~60 °C; ${\left[\alpha \right]}_{D}^{25}$ +78° (c 0.25, MeOH); UV (MeOH, λ_max_, nm) (logε): 262 (4.09); IR V_max_cm^−1^ (KBr): 3393, 2928, 1612, 1383, 1279, 1154, 1068, 988, 912; ^1^H and 13C-NMR data, see Table 1; ESI-MS *m*/*z*: 353 [M + Na]^+^; HR-ESI-MS at *m*/*z* 369.2039 [M + Na]^+^ (calcd for C_21_H_30_O_4_Na^+^ 369.2036).

Tricycloalternarene 2b (**2**): Yellowish amorphous powder; C_21_H_30_O_4_; m.p. 62~63 °C; ${\left[\alpha \right]}_{D}^{25}$ +75° (c 0.49, MeOH); UV (MeOH, λ_max_, nm) (logε): 263 (4.56, 200); IR V_max_cm^−1^ (KBr): 3420, 2920, 1720, 1650, 1618, 1455, 1380, 1310, 1267, 1205, 1170, 1150, 1080, 1040, 985, 915, 883, 830, 820; EI-MS *m*/*z*: 346 [M]^+^.

Tricycloalternarene 3a (**3**): Yellowish viscous oil; C_21_H_30_O_3_; ${\left[\alpha \right]}_{D}^{25}$ +74° (c 0.13, MeOH); UV (MeOH, λ_max_, nm) (logε): 262 (4.09); IR V_max_cm^−1^ (KBr): 3360, 2890, 1605, 1435, 1380, 1310, 1255, 1215, 1190, 1160, 1145, 1080, 1055, 1005, 950, 925, 915, 870, 820, 745; ESI-MS *m*/*z*: 353 [M + Na]^+^, 331 [M + H]^+^.

Tricycloalternarene 3b (**4**): Yellowish viscous oil; C_21_H_30_O_3_; ${\left[\alpha \right]}_{D}^{25}$ +73° (c 0.20, MeOH); UV (MeOH, λ_max_, nm) (logε): 264 (4.14); IR V_max_cm^−1^ (KBr): 3400, 2890, 1635, 1610, 1440, 1370, 1300, 1260, 1200, 1165, 1145, 1075, 980, 910, 875, 825, 745; ESI-MS *m*/*z*: 353 [M + Na]^+^, 331 [M + H]^+^.

ACTG-F (**5**): Yellowish viscous oil; C_21_H_30_O_5_; ${\left[\alpha \right]}_{D}^{25}$ +72° (c 0.23, MeOH); UV (MeOH, λ_max_, nm) (logε): 288 (5.50); IR V_max_cm^−1^ (KBr): 3400, 2920, 1720, 1680,1642, 1610, 1455, 1370, 1320, 1290, 1260, 1243, 1208, 1153, 1128, 1073, 1045, 992, 945, 922, 860, 805; ESI-MS *m*/*z*: 385 [M + Na]^+^.

### 3.4. Biological Assays

#### 3.4.1. Antimicrobial Test

Three human pathogenic strains, *E. coli* AB 94012, *S. aureus* AB 2010021 and *C. albicans* AY 204006, were purchased from the China Center for Type Culture Collection (CCTCC) and used as antimicrobial indicators in the study. Antimicrobial activity was assessed by the microbroth dilution method in 96-well culture plates developed by Patton and his colleagues [14]. Two positive controls, ampicillin and amphotericin B (Sigma-Aldrich, Buchs, Switzerland), were used as positive controls, and the solution of equal concentration of DMSO was made as a negative control. The bacteria were cultured in the LB medium for 24 h at 37 °C at 150 rpm, and the tested fungus was incubated in the PD medium for 48 h at 28 °C at the same rotatory speed. Bacterial cells or fungal spores were diluted to approximately 1 × 10^6^ CFU with PD or LB medium to evaluate the antimicrobial activities of pre-HPLC derived fractions and metabolites. Test solution at the initial concentration of 100 μM (100 μL) was added to 96-well microplate. Two-fold serial dilutions were made in the 96-well round-bottom sterile plates, and then 100 μL of the microbial suspension was added. After incubation, minimum inhibitory concentration (MIC) was taken as the lowest concentration of the test compounds in the wells of the 96-well plate in which the lowest microbial growth could be measured at 600 nm. All tests were carried out in triplicate.

#### 3.4.2. Cytotoxicity Test

Human lung adenocarcinoma A549 cell line (Shanghai Bioleaf Technology Co. Ltd., Shanghai, China) was grown in RPMI medium with 10% fetal bovine serum, penicillin (100 U/mL) and streptomycin (50 μg/mL) and cultured in a 96-well plate at a density of 5 × 10^5^ cells per well. All test compounds and positive control gefitinib (Sigma-Aldrich) were initially made up to 100 μM in DMSO. Each isolated compound was added to each well, respectively, with two-fold serial dilutions. Cell line without treatment by compound was used as the control. The incubation was performed in a humidified, 37 °C, 5% CO_2_-containing incubator for 24 h. Then 10 μL CCK-8 dye (Beyotime Institute of Biotechnology, Shanghai, China) was added to each well. Then the cell line was incubated at 37 °C for 2 h and plates were read in a Victor-V multilabel counter (Perkin-Elmer, Rodgau-Jügesheim, Germany) using the default europium detection protocol. IC_50_ value of each compound was calculated by comparison with DMSO-treated control wells and determined by the logit method from at least three independent tests [15].

## 4. Conclusions

This work reported firstly on the chemical investigation of one symbiotic strain *Aspergillus* sp. D from the coastal plant *E. chrysantha* Lindl. One new meroterpenoid tricycloalternarene 14b (**1**) and four known analogs, tricycloalternarenes 2b (**2**), 3a (**3**), 3b (**4**), and ACTG-toxin F (**5**) were isolated from the crude extract of fermentation broth and unambiguously determined on the basis of their spectroscopic spectra and optical rotation data, as well as by comparison with literature data. Bioassay results suggested that these metabolites had moderate cytotoxic effects on A549 cell line in vitro, with IC_50_ values range from 1.43 to 15.77 μM, and weak antimicrobial activities against *E. coli*, *S. aureus* and *C. albicans*, with MIC values of ≥15.63 μM. These findings indicated that the symbiotic strain D is one of versatile producers of bioactive tricycloalternarene derivatives with potential application in the field of medicine.

## Figures and Tables

**Table 1:** The ^1^H-NMR (500 MHz) and ^13^C-NMR (500 MHz) data of compound **1** (in CDCl_3_).

Position	1		2	
	δ_H_ (*J* in Hz)	δ_C_	δ_H_ (*J* in Hz)	δ_C_
1	1.29 (3H, s)	29.8	3.95 (2H, s)	68.7
2		70.7		135.2
2′	1.29 (3H, s)	29.8	1.62 (3H, s)	23.2
3	5.59 (H, d, 15.6)	139.6	5.25 (H, t, 7.6)	125.4
4	5.50 (H, m)	125.2	2.01 (2H, m)	24.9
5	1.97 (H, m) 2.23 (H, m)	27.8	1.50 (2H, m)	34.6
6	2.08 (H, m)	32.8	1.90 (H, m)	31.1
6′	0.96, (3H, d, 6.9)	19.4	0.96 (3H, d, 6.9)	13.7
7		149.7		150.0
8	5.33 (H, s)	120.4	5.34 (H, s)	119.9
9	2.43 (H, m) 2.60 (H, m)	44.9	2.36 (H, m) 2.60 (H, m)	45.1
10		88.3		88.8
10′	1.43 (3H, s)	23.4	1.45 (3H, s)	20.2
11	2.75 (H, m)	46.3	2.77 (H, m)	46.5
12	2.49 (H, m) 2.73 (H, m)	15.4	2.17 (H, m) 2.73 (H, m)	14.9
13		105.2		105.1
14		171.8		172.9
15	2.34 (H, m) 2.49 (H, m)	29.3	2.37 (m) 2.53 (m)	27.8
16	1.77 (H, m) 2.08 (H, m)	37.3	1.73 (H, m) 2.32 (H, m)	29.4
17	4.04 (H, dd, 12.9, 5.4)	71.6	4.02 (H, dd, 12.9, 5.4)	71.0
18		197.8		197.8

## Supplementary Materials

1D and 2D NMR spectra, and HR-ESI-MS spectrum for compound **1**, ^1^H and 13C-NMR spectra for **2**, 1H-NMR spectra for **3**–**5**, and LR-ESI-MS spectra for **2**–**5** are available online.
