# Supplementary material for: Tricycloalternarene Analogs from a Symbiotic Fungus Aspergillus sp. D and Their Antimicrobial and Cytotoxic Effects
Source: Molecules. 2018 Apr 9;23(4):855. doi: 10.3390/molecules23040855 (PMC6017176; doi:10.3390/molecules23040855)
Supplement: Supplementary file 1 [file molecules-23-00855-s001.pdf]

Supplementary material for

# Tricycloalternarene analogs from a symbiotic fungus *Aspergillus* sp. D and their antimicrobial and cytotoxic effects

Huawei Zhang, Ziping Zhao, Jianwei Chen, Xuelian Bai and Hong Wang *

## Table of Contends


| | | |
|---|---|---|
| Figure S1 | $^1$H NMR spectrum of **1** | S2 |
| Figure S2 | $^{13}$C NMR spectrum of **1** | S3 |
| Figure S3 | HR-ESI-MS spectrum of **1** | S4 |
| Figure S4 | HSQC spectrum of **1** | S5 |
| Figure S5 | HMBC spectrum of **1** | S6 |
| Figure S6 | $^1$H-$^1$H COSY spectrum of **1** | S7 |
| Figure S7 | NOESY spectrum of **1** | S8 |
| Figure S8 | $^1$H NMR spectrum of **2** | S9 |
| Figure S9 | $^{13}$C NMR spectrum of **2** | S10 |
| Figure S10 | LR-ESI-MS spectrum of **2** | S11 |
| Figure S11 | $^1$H NMR spectrum of **3** | S12 |
| Figure S12 | LR-ESI-MS spectrum of **3** | S13 |
| Figure S13 | $^1$H NMR spectrum of **4** | S14 |
| Figure S14 | LR-ESI-MS spectrum of **4** | S15 |
| Figure S15 | $^1$H NMR spectrum of **5** | S16 |
| Figure S16 | LR-ESI-MS spectrum of **5** | S17 |

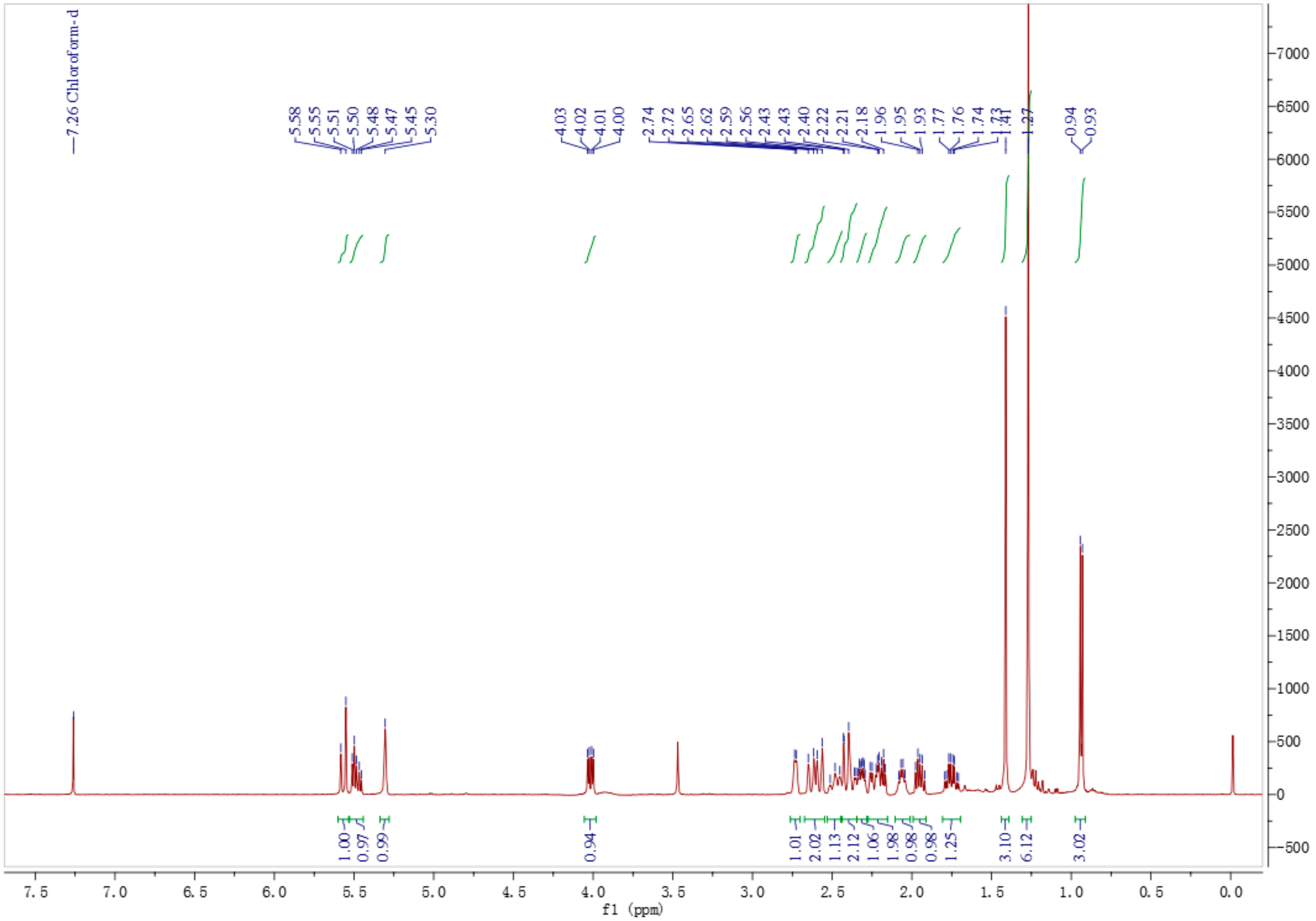


Figure S1. $^{1}$H NMR spectrum of **1**.

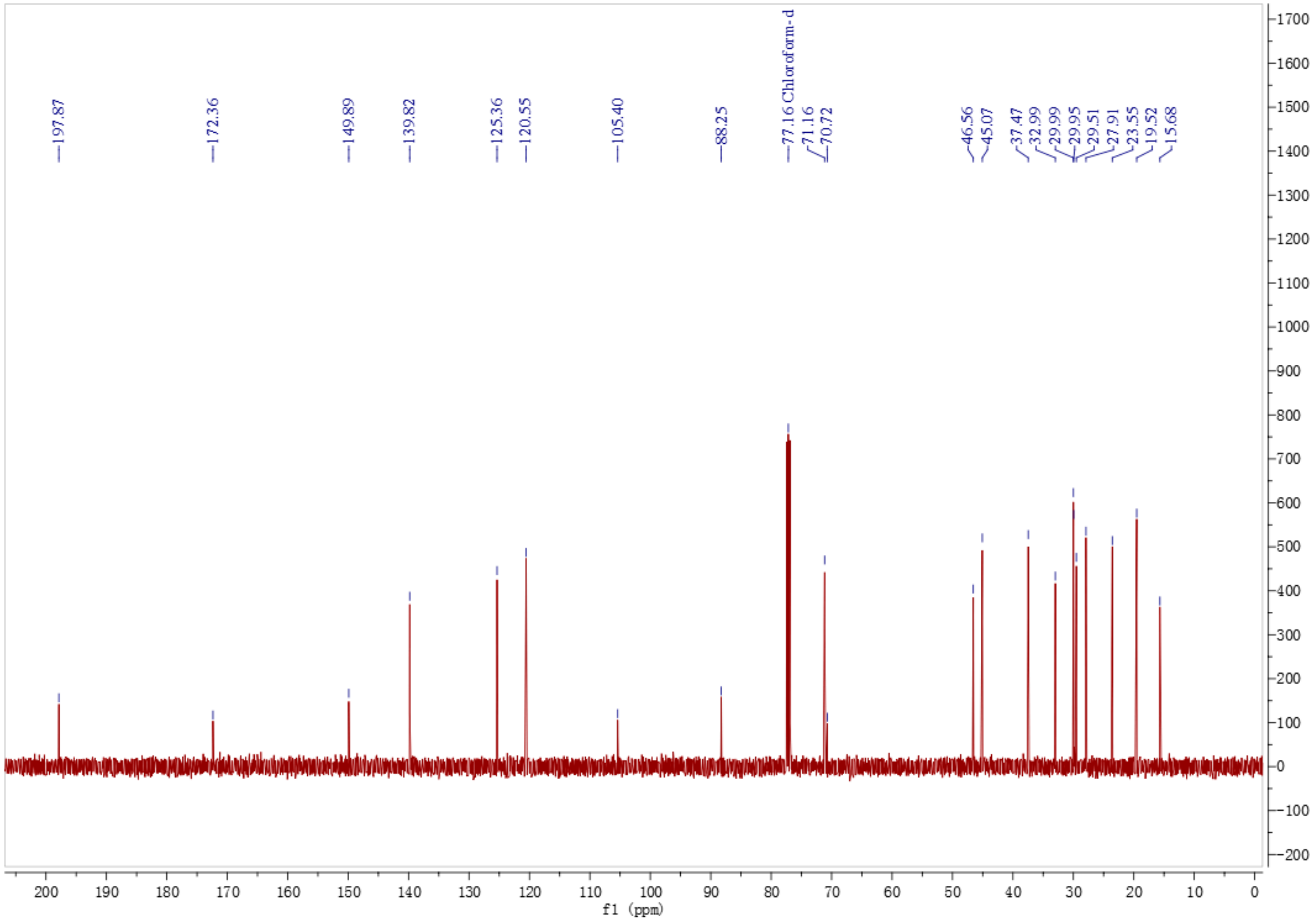


Figure S2. $^{13}$C NMR spectrum of **1**.

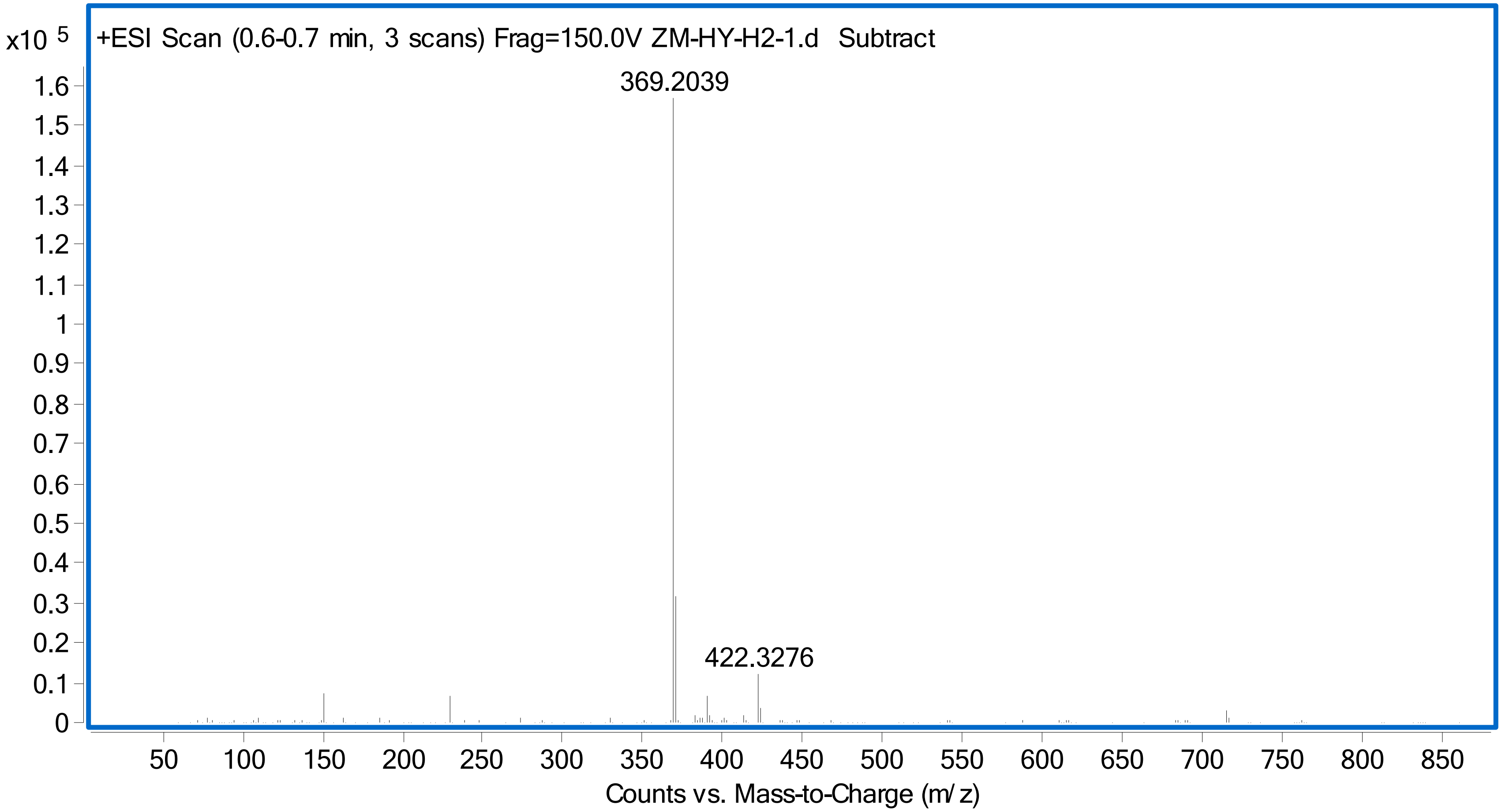


Figure S3. HR-ESI-MS spectrum of **1**.

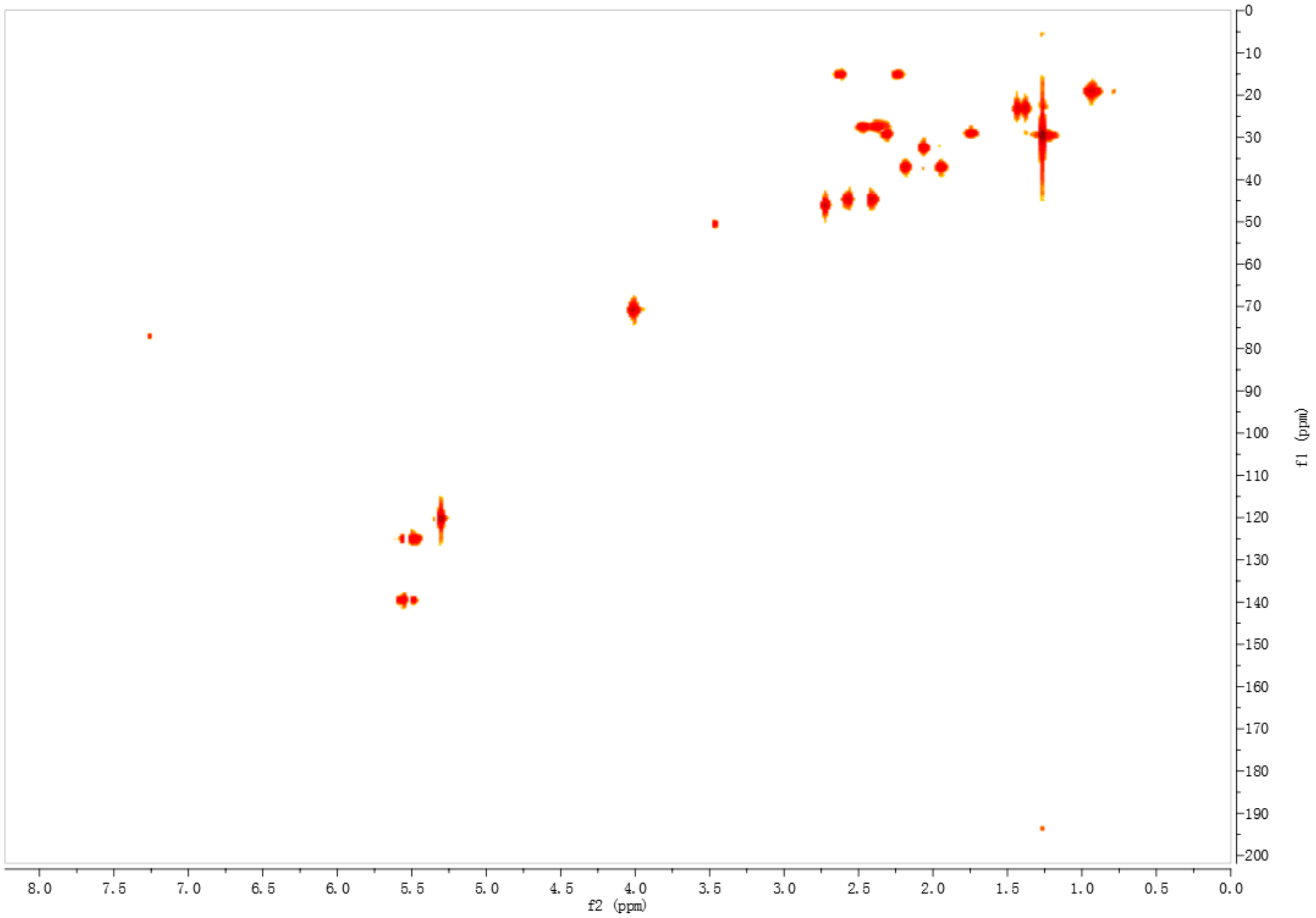


Figure S4. HSQC spectrum of **1**.

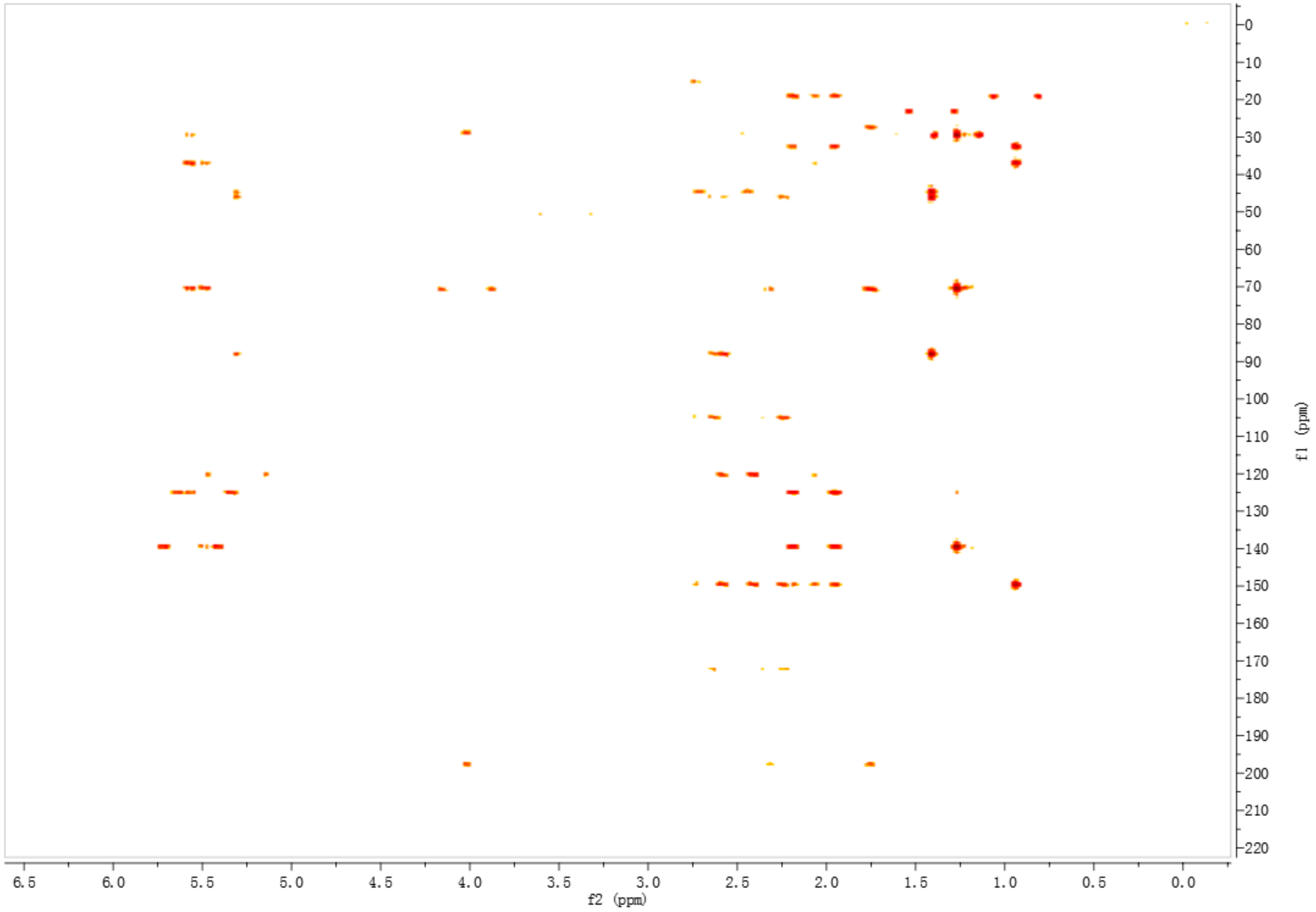


Figure S5. HMBC spectrum of **1**.

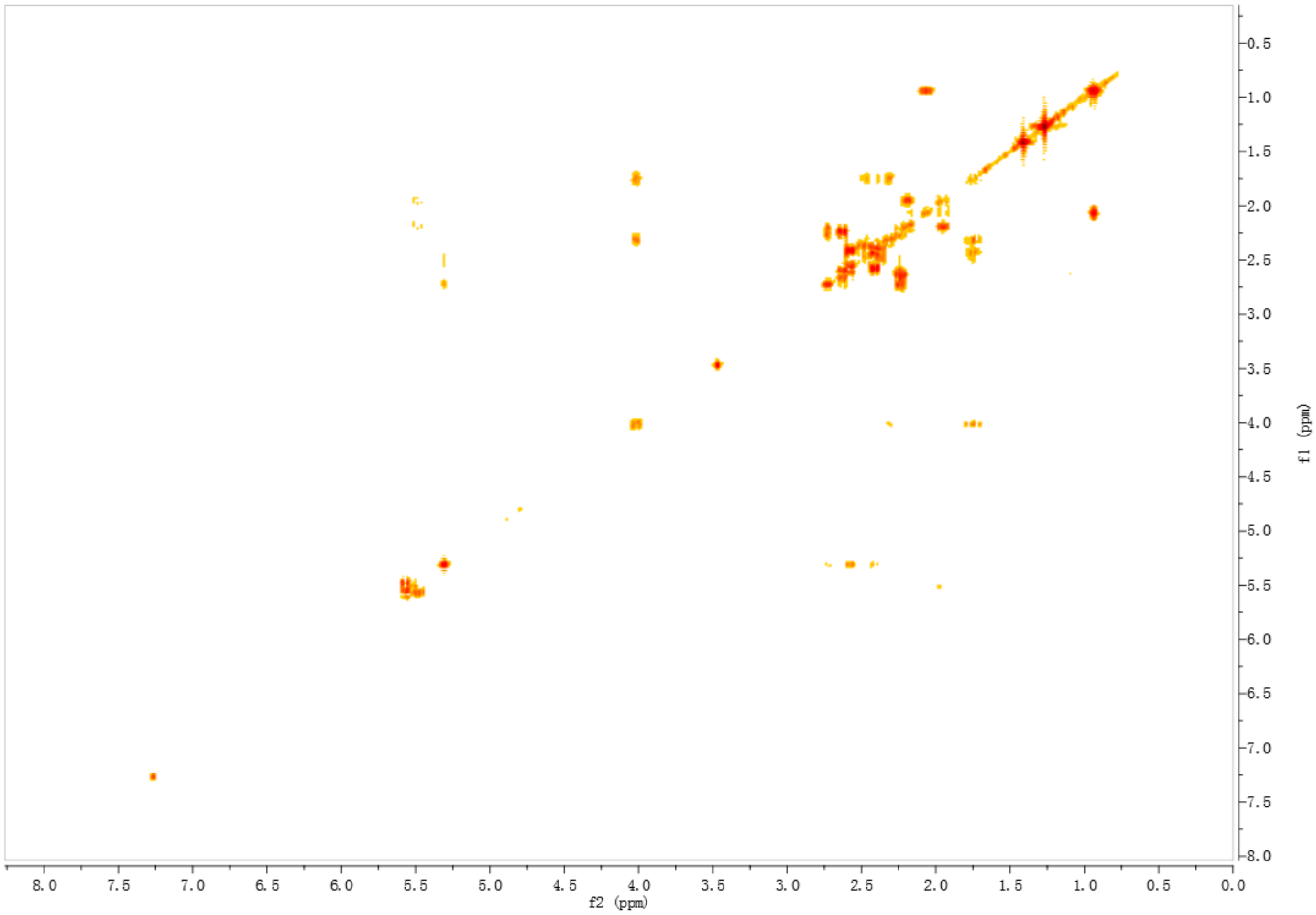


Figure S6. $^{1}H$-$^{1}H$ COSY spectrum of **1**.

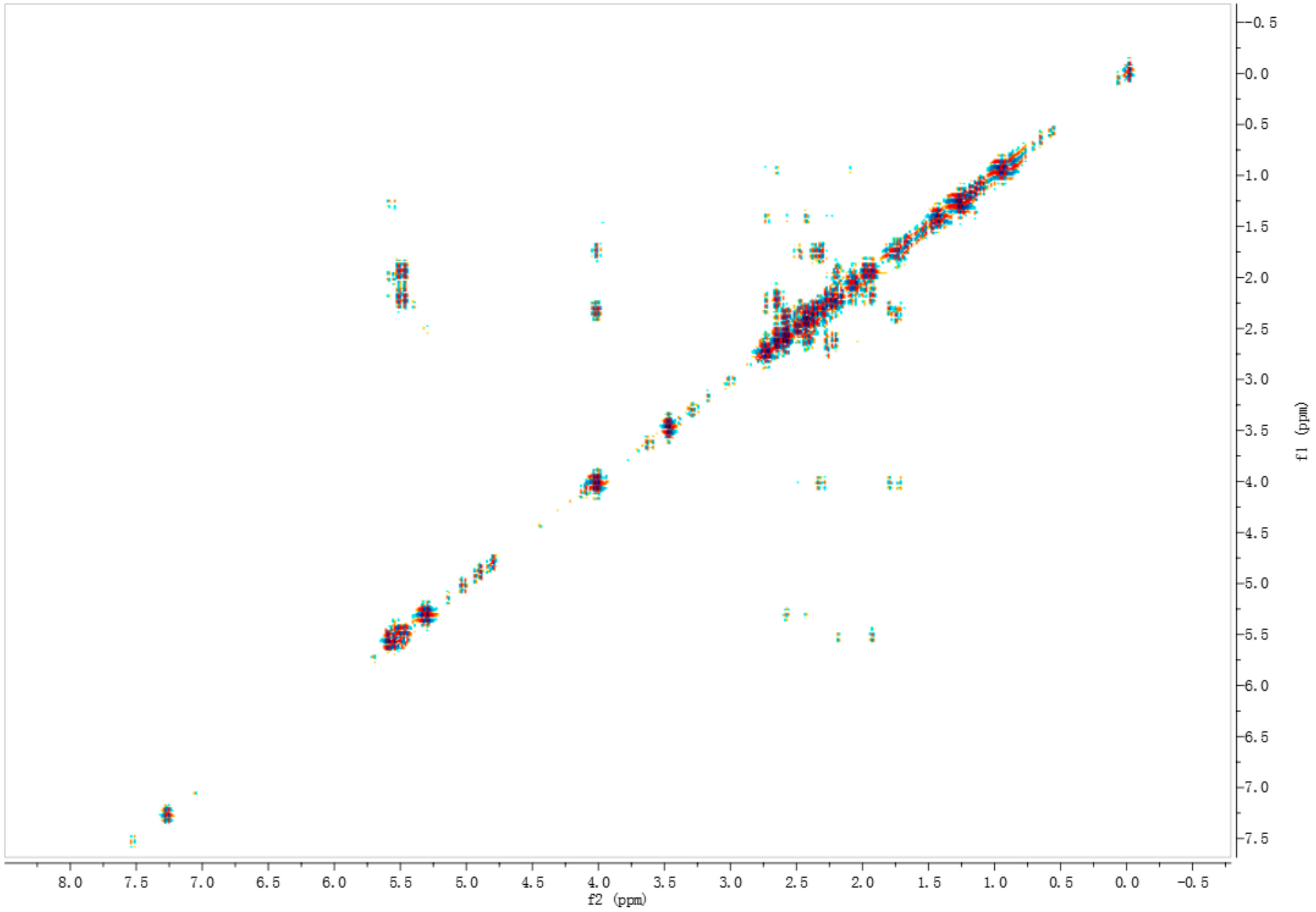


Figure S7. NOESY spectrum of **1**.

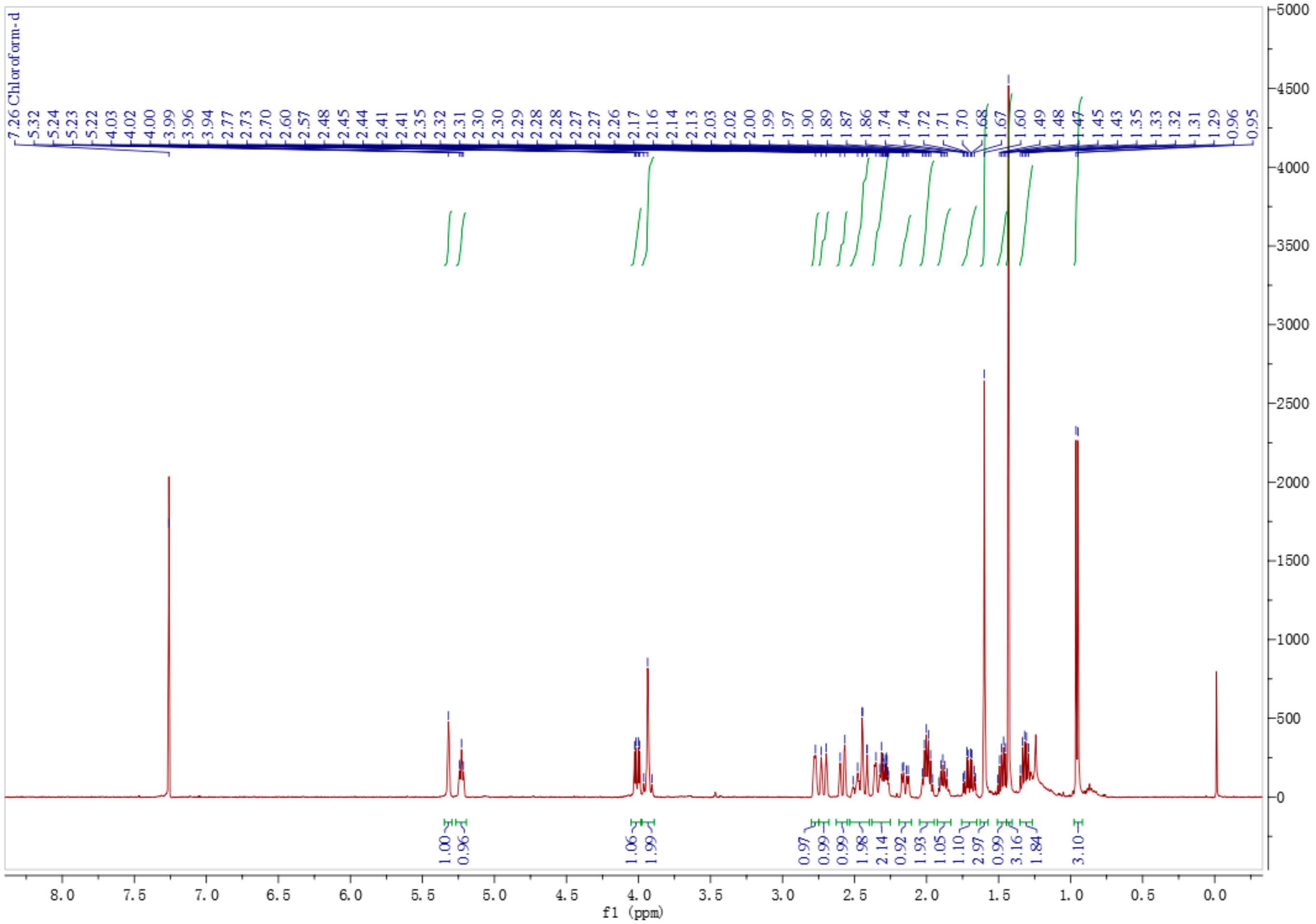

Figure S8. $^{1}H$ NMR spectrum of **2**.

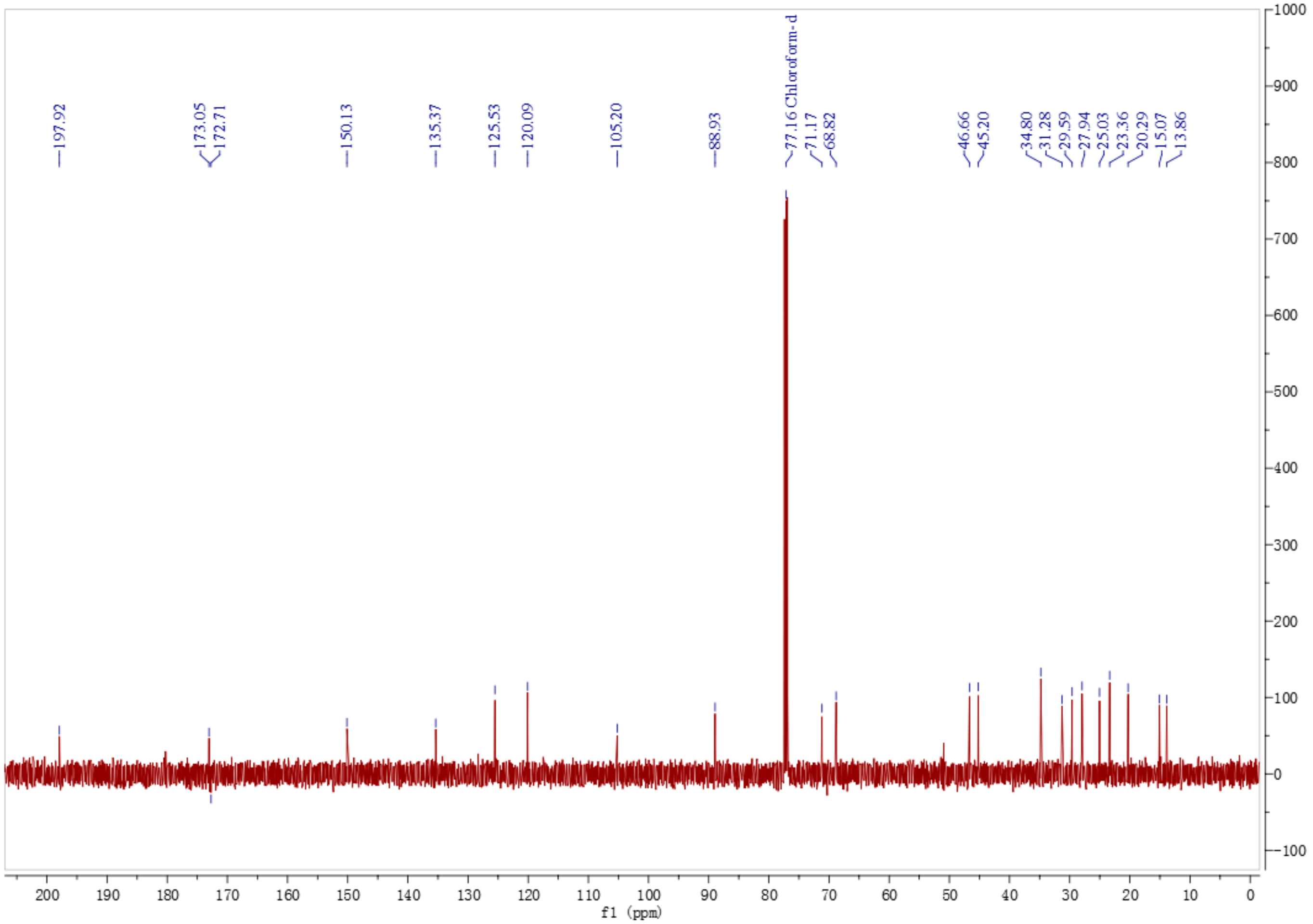


Figure S9. $^{13}$C NMR spectrum of **2**.

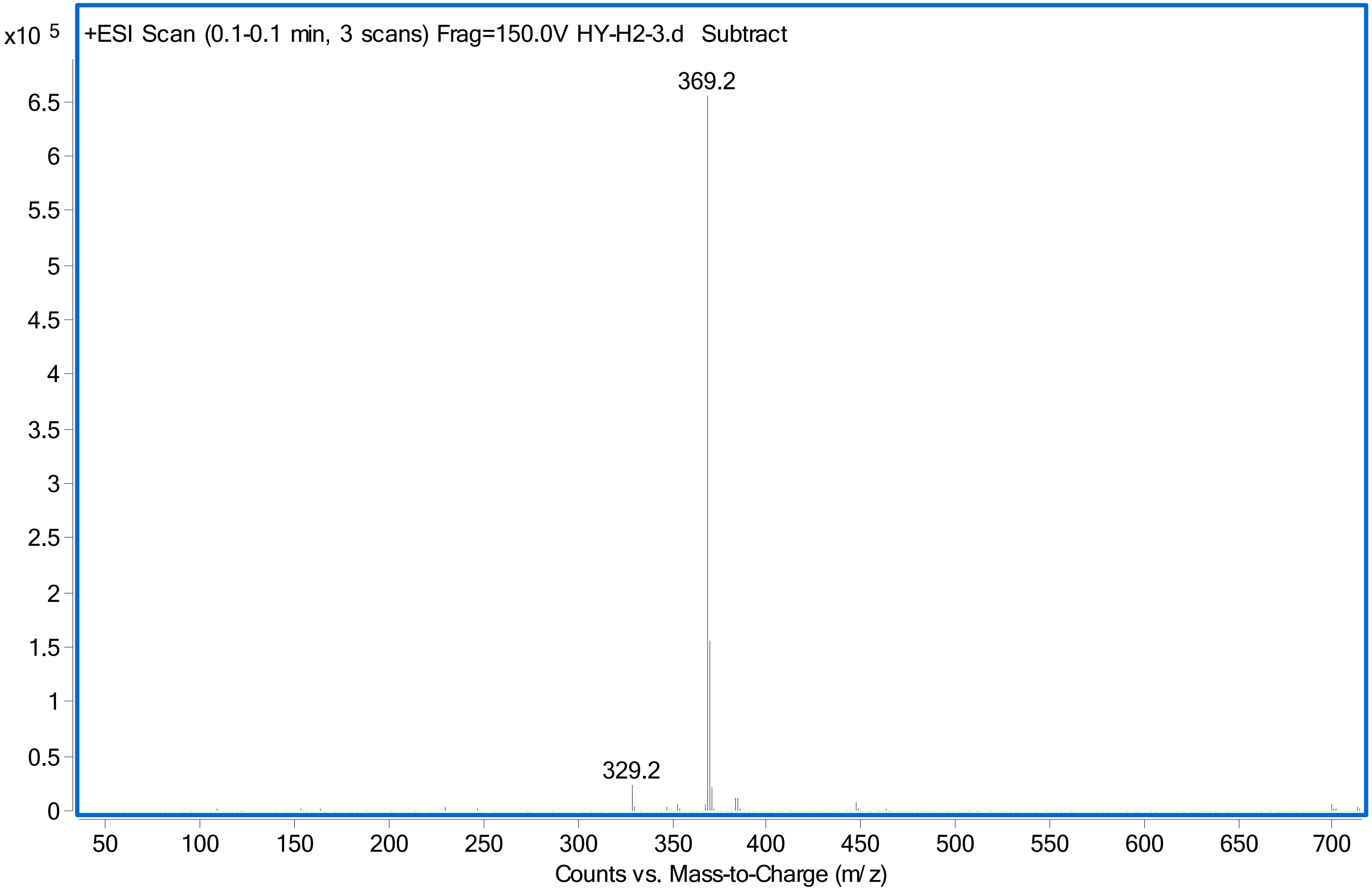


Figure S10. LR-ESI-MS spectrum of **2**.

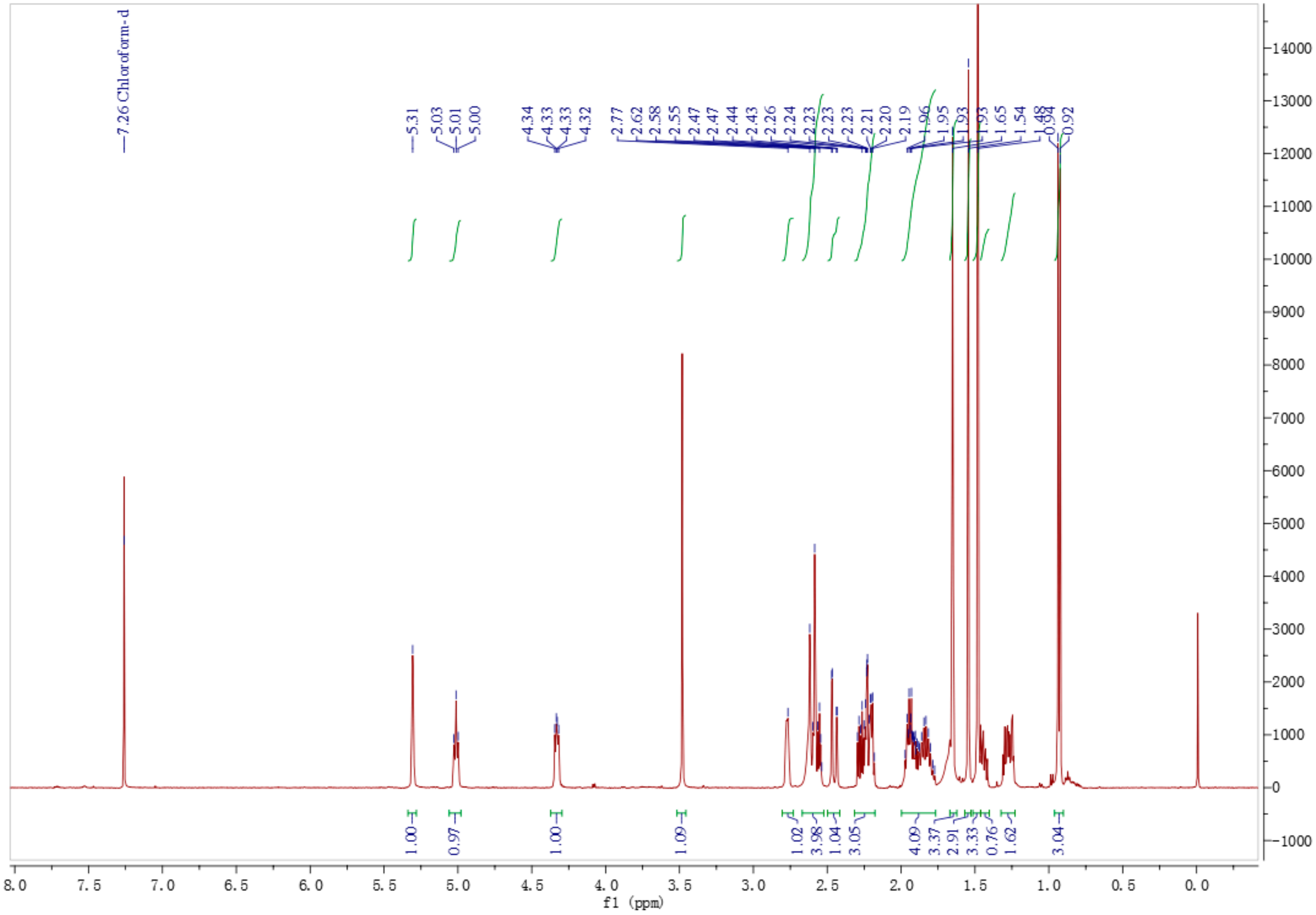

Figure S11. $^{1}H$ NMR spectrum of **3**.

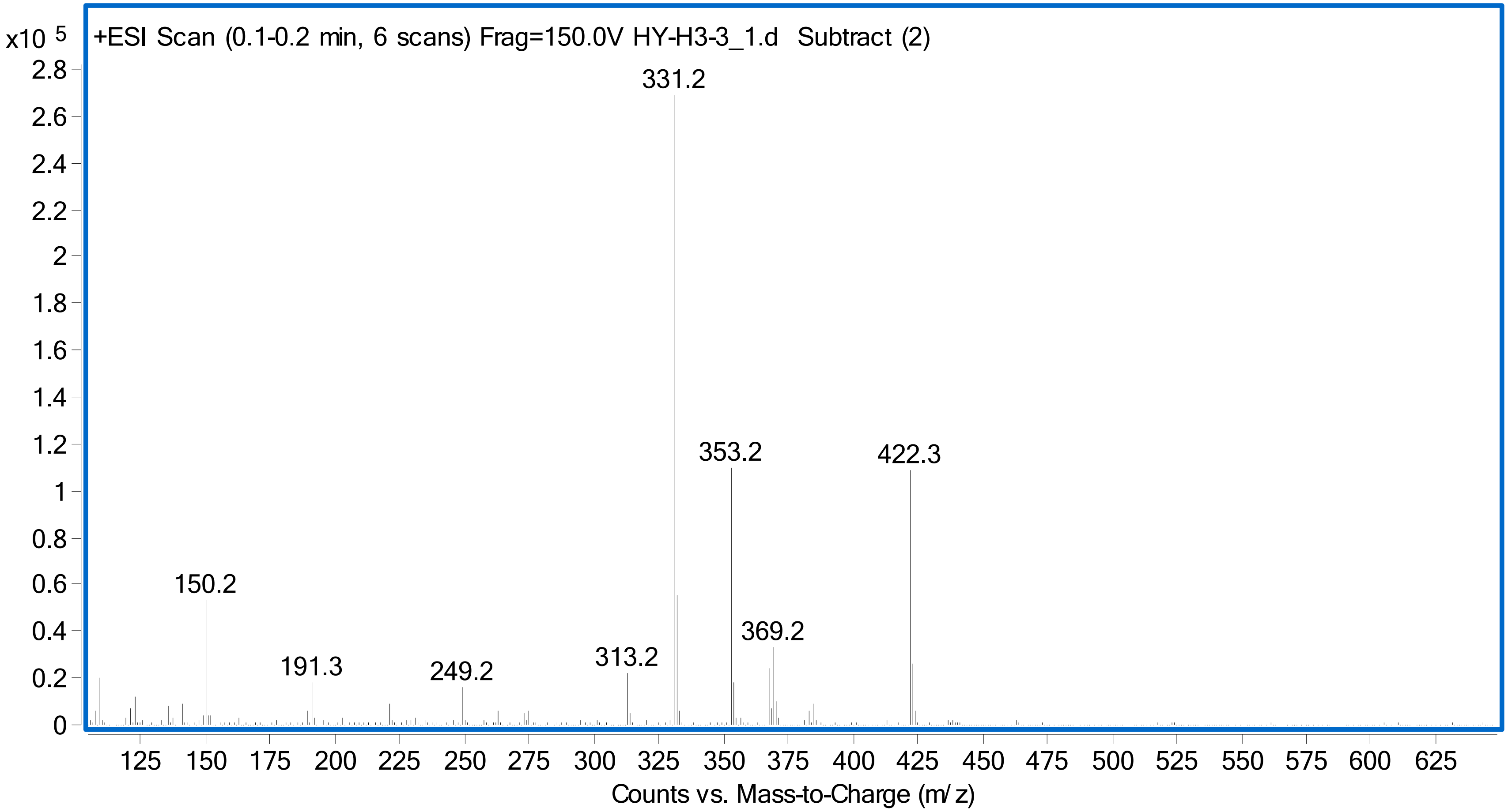


Figure S12. LR-ESI-MS spectrum of **3**.

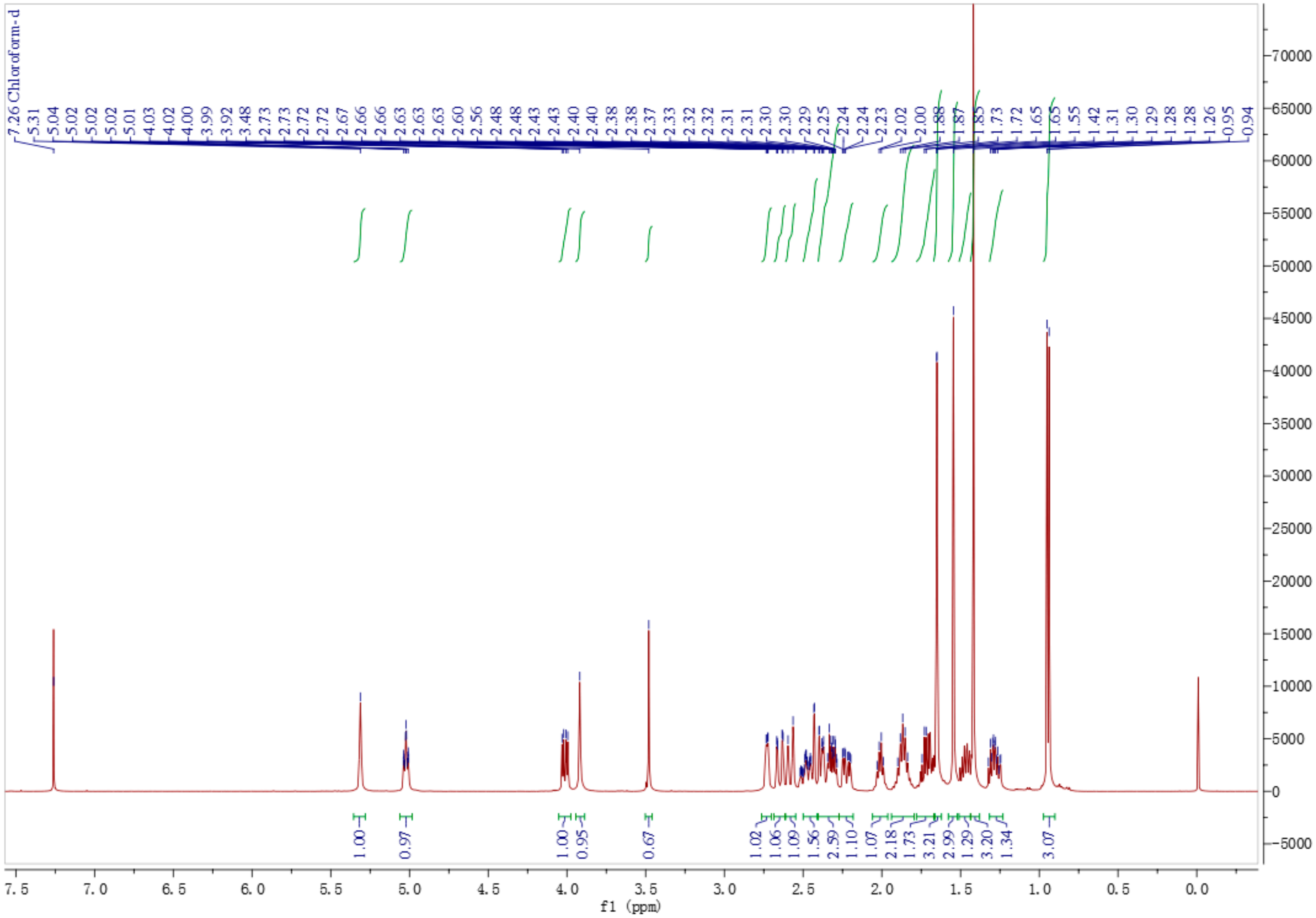

Figure S13. $^{1}H$ NMR spectrum of **4**.

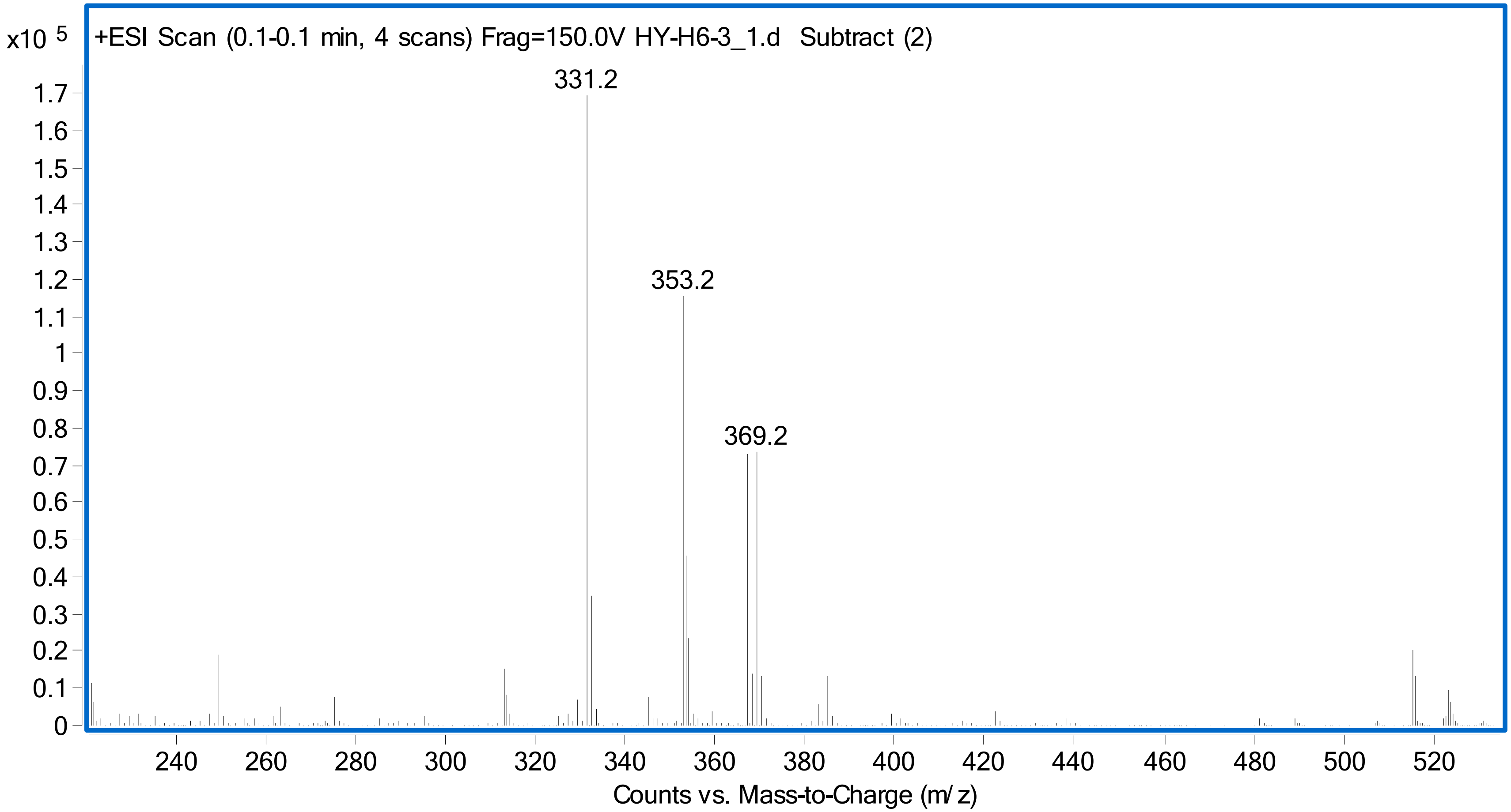


Figure S14. LR-ESI-MS spectrum of **4**.

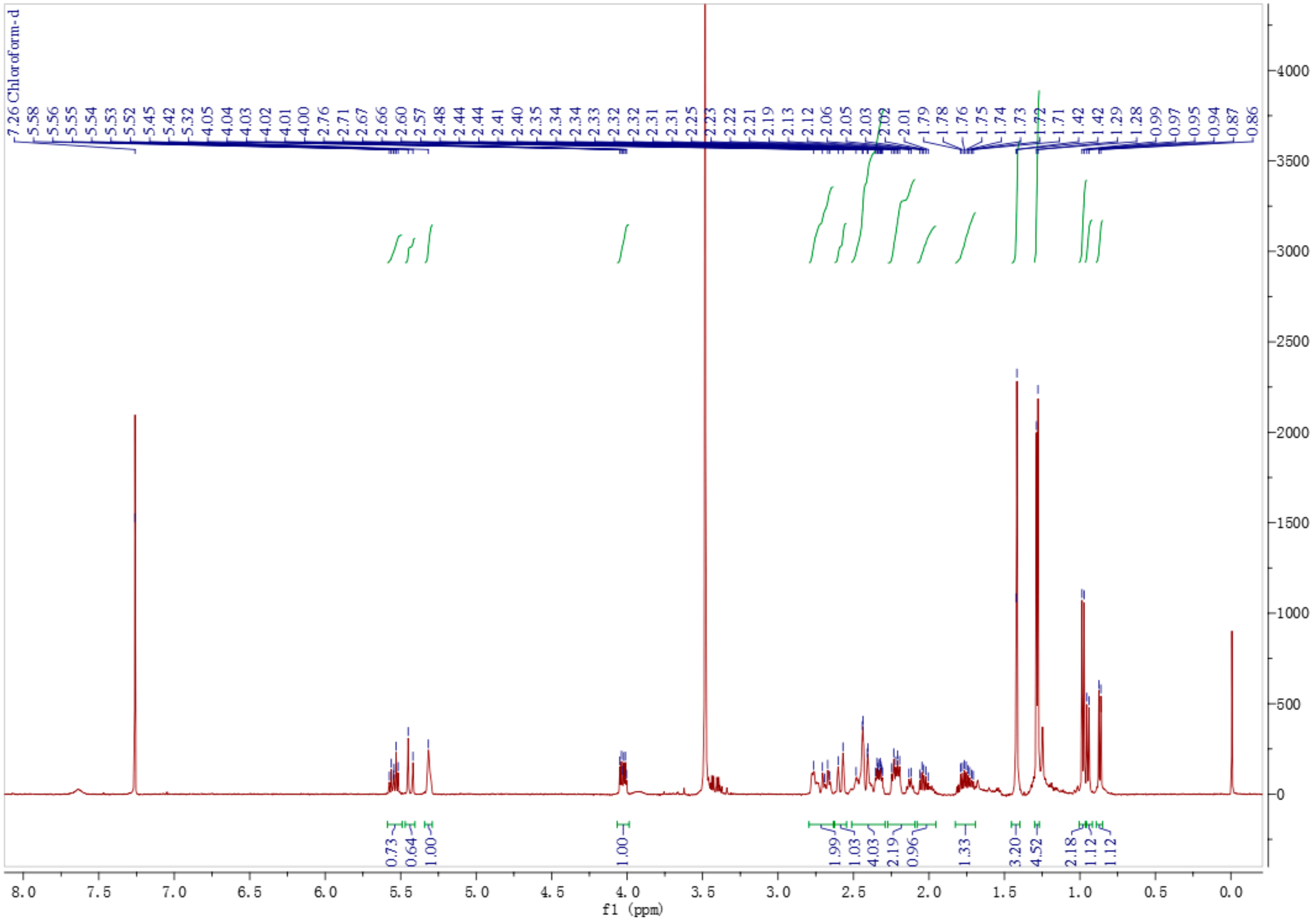

Figure S15. $^{1}H$ NMR spectrum of **5**.

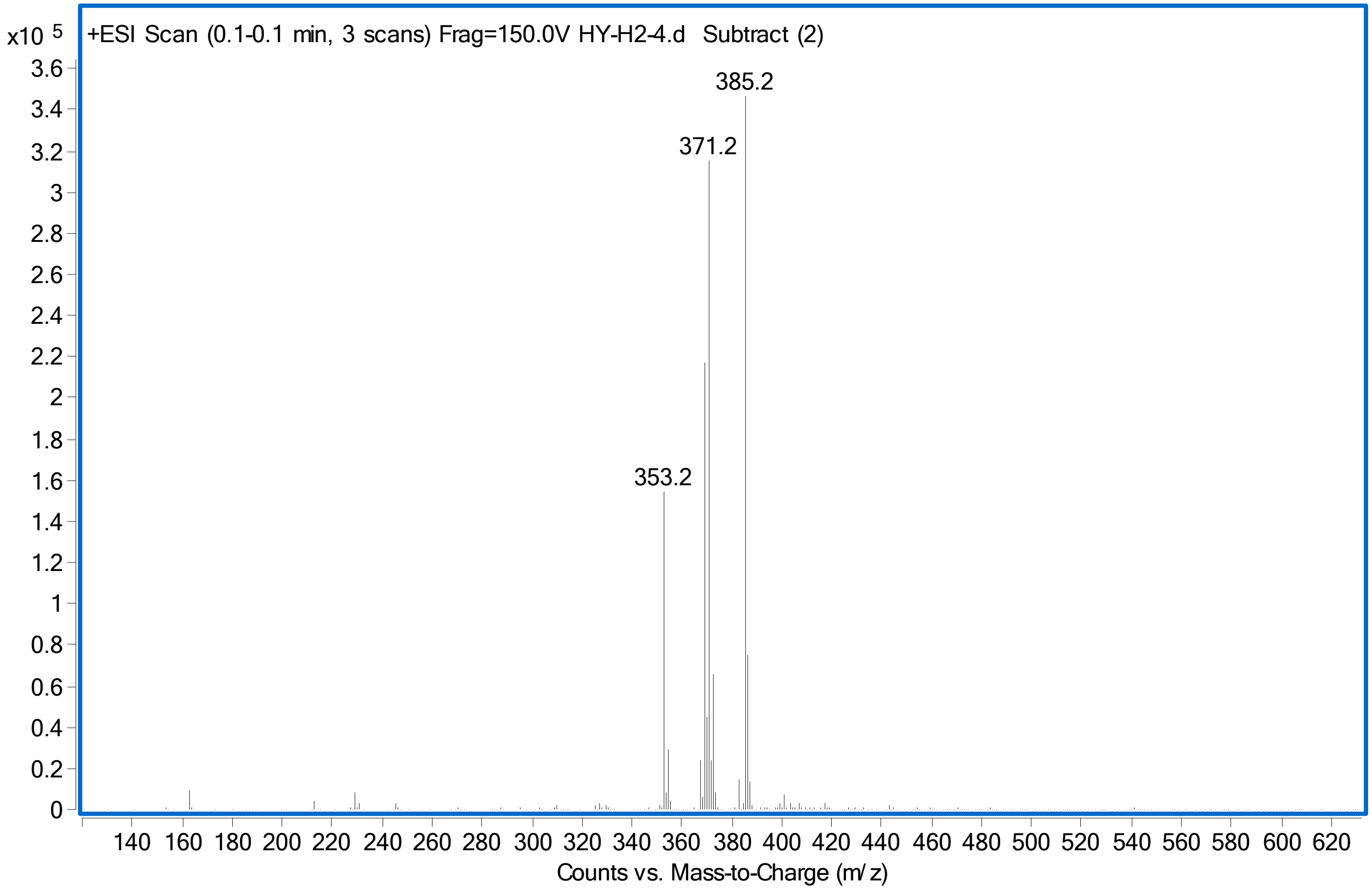


Figure S16. LR-ESI-MS spectrum of **5**.
